# Who Buys Products with Nutrition and Health Claims? A Purchase Simulation with Eye Tracking on the Influence of Consumers’ Nutrition Knowledge and Health Motivation

**DOI:** 10.3390/nu11092199

**Published:** 2019-09-12

**Authors:** Johann Steinhauser, Meike Janssen, Ulrich Hamm

**Affiliations:** 1 Department of Agricultural and Food Marketing, Faculty of Organic Agricultural Sciences, University of Kassel, Steinstr. 19, 37213 Witzenhausen, Germany; j.steinhauser@uni-kassel.de (J.S.); hamm@uni-kassel.de (U.H.); 2 Department of Management, Society and Communication, Copenhagen Business School, Dalgas Have 15, 2000 Frederiksberg, Denmark

**Keywords:** health claims, nutrition knowledge, eye tracking, visual attention, consumer behavior, purchase decision

## Abstract

Nutrition and health claims are seen as a way of promoting healthy aspects of food. However, the results of previous studies have been contradictory regarding the effect of these claims on purchase. This study aims to achieve a better understanding of how the consumer characteristics ‘nutrition knowledge’ and ‘health motivation’ influence the purchase of products with nutrition and health claims and what role gaze behavior plays. We included gaze behavior in our analysis, as visual attention on the claims is a precondition to its influence on the purchase decision. In a close-to-realistic shopping situation, consumers could choose from three-dimensional orange juice packages labeled with nutrition, health, and taste claims. In total, the sample consisted of 156 consumers. The data were analyzed with a structural equation model (SEM), linking the purchase decision for products with claims to gaze data recorded with a mobile eye tracker and consumer and product-related variables collected via the questionnaire. Results showed that the variables in the SEM explained 31% (8%) of the variance observed in the purchase of products with a nutrition (health) claim. The longer a consumer looked at a specific claim, the more likely the consumer would purchase the respective product. The lower the price and the higher the perceived healthiness and tastiness of the product further heightened its likelihood of being purchased. Interestingly, consumers with higher nutrition knowledge and/or higher health motivation looked longer at the nutrition and health claims; however, these consumer characteristics did not show an effect on the purchase decision. Implications for policy makers and marketers are given.

## 1. Introduction

In today’s grocery stores, consumers encounter a great variety of food products and their packages are full of information. Manufacturers want their food products to attract consumers’ attention [1]. Since the interest in leading a healthy lifestyle has been growing [2,3], the use of nutrition and health claims seems promising for manufacturers. Such claims link the food product to healthiness by stating positive nutritional characteristics or naming an explicit health benefit of the nutrients it contains. Around one third of the products in grocery stores are labeled with nutrition and health claims [4,5,6,7,8].

Research on nutrition and health claims has shown both positive and negative effects for these claims on consumers’ preferences and purchase behavior [9,10,11,12,13,14,15,16,17]. The discrepancy in the effects reported by previous studies has been commented on by other authors [18,19,20]. They have suggested that an explanation for the discrepancy might be that different consumer groups react differently to nutrition and health claims. The characteristics of consumers might influence the effect of nutrition and health claims on their preferences and/or purchase behavior and should be included in future research [21,22,23,24]. Several recent articles have pointed to consumers’ nutrition knowledge and health motivation as promising independent variables for future research [9,10,13,21,23,25,26,27].

Nutrition knowledge is defined as a “scientific construct that nutrition educators have created to represent individual’s cognitive processes related to information about food and nutrition” [28]. Health motivation is defined as “consumers’ goal-directed arousal to engage in preventive health behaviors” [29]. Earlier studies have shown that consumers with higher nutrition knowledge [30,31] or higher health motivation [32,33] stated they read claims more often than those less knowledgeable and motivated. Consumers with higher health motivation had a higher purchase intention and likelihood of choosing products with nutrition or health claims [25,34,35]. However, other studies have shown no influence of nutrition knowledge [36] or health motivation [37,38] on the purchase intention for these products. The present study went one step further by including both of these two consumer characteristics in a close-to-realistic shopping experiment, and by analyzing their influence on actual purchasing behavior for products with nutrition and health claims.

In addition, we took into consideration the fact that nutrition knowledge and health motivation might influence consumers’ visual attention towards nutrition and health claims during their purchase decision. Previous studies have shown that motivation influences visual attention on food packaging. A higher motivation towards healthy living or a higher product involvement mostly showed an increase in visual attention on certain package labels [39,40,41,42,43,44]. The effect of topic-relevant knowledge on visual attention has not been investigated in the context of food like it has been in other fields such as art, chess, or sports [45,46,47,48]. Before consumers decide to purchase a food product, they normally look at the product they are going to purchase; thus, visual attention usually precedes the purchase decision [49,50,51,52]. Previous research has shown that visual attention influences food choice, in that the more visual attention a package or a certain label on a package receives, the more likely it is that this product will be chosen [44,51,52,53,54,55,56,57]. The eye movements, and consequently the visual attention to stimuli, can be measured with an eye tracking device. In the present study, head mounted eye tracking glasses were used to ensure that consumers were able to act naturally in front of a shopping shelf with three-dimensional food packages.

Besides consumer characteristics, the food product’s attributes also influence shopping for food. Therefore, the most important product attributes were incorporated into the study. According to previous research, these are price, brand, perceived tastiness, and the healthiness of the products [58,59,60,61,62].

The overall aim of the present work was to analyze the factors which influence the purchase decision for food products with nutrition and health claims. The main research questions were the following:

(1) What effects do consumers’ nutrition knowledge and health motivation have on the purchase decision for products labeled with nutrition and health claims?
(2a) What effects do consumers’ nutrition knowledge and health motivation have on visual attention on food packages?
(2b) How does visual attention on claims mediate the effect of nutrition knowledge and health motivation on the purchase decision?
(3) What effects do price, brand, perceived tastiness, and healthiness have on the purchase decision for products labeled with nutrition and health claims?

The study is innovative because it has gone beyond previous survey-based research on claims. With the use of head-mounted eye tracking glasses, this purchase simulation analyzed the influence of nutrition knowledge and health motivation on gaze behavior, and the influence of these three constructs on the purchase decision. Typical product attributes influencing the purchase for food were incorporated in the study. The data were analyzed with a structural equation model.

In a recent review article on nutrition and health claims, the authors concluded that the studies conducted in more natural settings indicated that nutrition and health claims might play a much smaller role than studies conducted in more artificial settings would suggest [18]. Therefore, these authors, along with others, have advocated for researching the effects of nutrition and health claims on actual behavior with real three-dimensional packages in a purchase situation embedded in a more realistic environment [13,63,64]. In the present study, we followed these recommendations and tested the nutrition and health claims in a close-to-realistic shopping experiment.

## 2. Theoretical Framework

According to the Elaboration-Likelihood Model (ELM) of Petty and Cacioppo [65], consumers’ motivation and ability influence the elaboration of information [66,67,68]. Consumers’ ‘motivation’ comprises the personal relevance to the information’s topic, while ‘ability’ comprises the topic-relevant knowledge of the consumer [65]. Additionally, the visual attention towards information can be incorporated into the ELM [69]. Firstly, visual attention naturally precedes the elaboration of information such as package labels and is an indicator for the elaboration of the information which is gazed at [70,71,72,73,74,75,76]. Secondly, motivation and knowledge are factors known to influence consumers’ visual attention [1,43,50,51,77,78,79]. Furthermore, previous research has shown that visual attention is a precondition to making purchase decisions [52,53]. Overall, visual attention mediates between the two consumer characteristics; ‘motivation’ and ‘knowledge’ on one side, and ‘purchase behavior’ on the other.

While gazing at a product package, consumers use both internal and external information. At the point of sale, the external information the consumers can use is limited to the labels on the package, such as the ingredient list or nutrition and health claims [80,81]. The internal information is the knowledge of the consumer about product-specific attributes [11]. Pioneer studies have shown that consumers with higher topic-relevant knowledge process and interpret information differently [66,67,82,83,84]. Topic-relevant knowledge on its own does not necessarily lead to a determined behavior. For example, knowing that certain eating habits are unhealthy might not result in giving them up [85]. However, consumers who are more motivated, for example, to lead a healthier lifestyle might be more inclined to change their behavior. Thus, motivation and knowledge are usually seen as two closely related constructs [66,81,86,87]. Research has also shown that motivation influences consumers’ engagement and time spent searching for information [10,88,89,90].

ELM has been applied in many research studies on the influence of food labeling on consumer behavior with the consumer characteristics ‘nutrition knowledge’ and ‘health motivation’ representing ‘ability’ and ‘motivation’ [29,91]. ‘Nutrition knowledge’ and ‘health motivation’ are the key variables which influence the processing of information on food packages, especially the information related to nutrition and health such as nutrition labels or nutrition and health claims [63,64,80,87,92,93].

**Figure 1:**
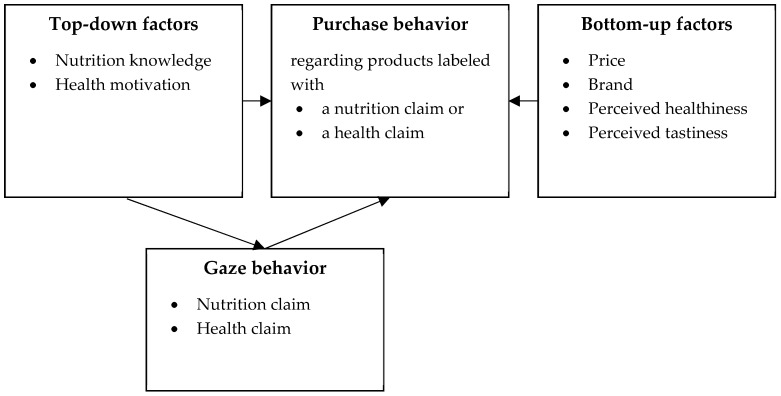
Conceptual model of the study.

The designated roles of nutrition knowledge and health motivation as part of the ELM, with their influence on visual attention and purchase decision, can be comprised under the term ‘top-down factors’, which represent the characteristics of the consumer. Accordingly, there are also ‘bottom-up factors’ representing the characteristics of the product [94], which include consumers’ perception of the product guided by its characteristics and attributes [95]. In the present study, we included the following bottom-up factors in the analysis: Price, brand, perceived tastiness, and healthiness, as these aspects are among the most important factors for the purchase of food [59,60,62]. The conceptual model of this study is depicted in Figure 1.

## 3. Methodology

### 3.1. Eye Tracking

With eye tracking, certain limitations of conventional research methods can be overcome, such as the limited ability of consumers to remember what they paid attention to during the purchase process or the unwillingness of consumers to disclose certain information. In research on nutrition and health claims, consumers might estimate the attention paid to these claims wrongly in post-purchase questionnaires, whereas eye tracking shows directly how long consumers visually attend to these claims [52]. Although eye tracking is an objective method of measuring the visual attention of consumers [41], it cannot explain why consumers looked at certain product elements [48,50,52,69]. An additional interview with the consumers could provide information about the underlying reasons for the gaze behavior of consumers. Hence, the combination of an eye tracking task with a subsequent questionnaire seems promising [48,50,52].

A head-mounted eye tracking system was chosen for this present study because it expands the use of eye tracking into far more true-to-life surroundings than a stationary eye tracking system with a monitor or a wall projection [48,52,69]. Previous eye tracking research yielded differences in the gaze behavior between the two different systems [96,97]. The application of a head-mounted eye tracking system is more appropriate for measuring gaze behavior in a shopping experiment for food. With the use of a head-mounted eye tracking system in this present study, participants were able to move freely in front of a shopping shelf, look at the products from different angles, and take products off the shelf for closer inspection.

### 3.2. Study Design and Stimuli

When the participants entered the laboratory (one-by-one), they were briefed about the shopping task in the laboratory’s simulated grocery store. After the successful calibration of the eye tracking system (SMI Eye Tracking Glasses 2 Wireless, 60 Hz), the interviewer proceeded by reading the task instructions to the participant. The participants were told to imagine they were going shopping for orange juice and to buy one of the orange juices offered. Afterwards, they would pay with their own money. The participants were instructed to choose the product they would purchase in a normal shopping situation. Further, they were told to take as much time for their shopping as they would usually need. In the present study, the briefing of participants was deemed important, as other authors have emphasized that giving a task to the participants of an eye tracking experiment is necessary to prevent participants not only from guessing the purpose of the experiment, but also from looking aimlessly at the stimuli without knowing what to do, rendering the patterns of the participants’ gaze behaviors impossible to compare [48,50].

During the shopping simulation, the participants stood in front of a shopping shelf filled with three brands of orange juices. Each participant was told to purchase one brand. To make the experiment look as realistic as possible, the stimuli were three-dimensional food packages with real brands. To eliminate the influence of well-known brands on the product choice and thus habitual purchase decisions, brands from another German speaking country (Austria) were chosen for the shopping task. The nutrition and health claims were well-incorporated into the package design to avoid any forced exposure. One product alternative was labeled with the nutrition claim, another alternative with the health claim, and a third alternative with a taste claim. Offering one alternative labeled solely with a taste claim (‘simply delicious’) is common practice in research on nutrition and health claims, as it counters the mere label effect [98,99,100,101,102,103]. The three claims ‘nutrition’, ‘health’, and ‘taste’ were rotated among the three product brands across the sample. Also, three price levels were rotated among the three product brands. All the other product attributes such as the nutrition table, ingredient list, etc. were made identical among the three brands. The tested nutrition and health claims conformed, in content, wording, and use for the product category orange juice, to European Union (EU) regulations No. 1924/2006 Art. 5 par. 1.b. and EU Regulation No. 1169/2011 annex XIII part A and listed in the EU Register of nutrition and health claims made on foods [104]. The nutrition and health claims are shown in Table 1. All subjects gave their informed consent for inclusion before they participated in the study. The study was conducted in accordance with the ethical standards defined in the 1964 Helsinki Declaration and the study design was approved by the university authorities. No data were collected that could reveal the identity of the participants.

After the participants finished their purchase, the eye tracking glasses were taken off and the participants filled out a self-administered computer-assisted interview. Finally, the participants were debriefed and given their remuneration.

### 3.3. Measures & Variables

The constructs of the conceptual model and their indicators are shown in Table 2.

### 3.4. Participants

All participants were recruited in a medium-sized city in central Germany (Kassel) with average purchase power (Table 3). The recruiters were positioned at predefined spots in the pedestrian area of the city’s main shopping promenade. They systematically approached every third person passing by, resulting in a random sample. To further ensure a representative sample of shoppers, the recruitment took place every day of the week and during the whole daytime. In order to take part in the study, the individuals approached had to fulfill two screening criteria, i.e., they had to go grocery shopping at least occasionally, and they had to purchase orange juice at least occasionally. A remuneration of €10 was offered for participating in the experiment. There was no limitation regarding the recruitment of participants with impaired vision because SMI’s optical lenses could be attached to the eye tracking glasses. At no time did the recruiters reveal the purpose of the study. Instead, they provided a vague cover story of a shopping task for food. The recruitment yielded a sample of 156 participants usable for further analyses whose characteristics are displayed in Table 3.

### 3.5. Data Analysis

**Figure 2:**
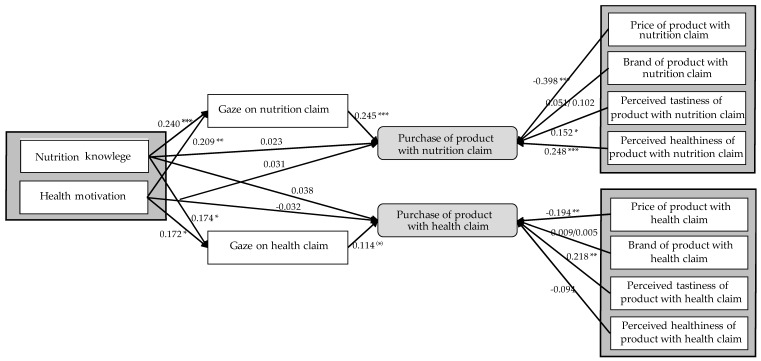
The structural equation model, including its path coefficients and their significance. Significance *p* < 0.001 = ***; *p* < 0.01 = **; *p* < 0.05 = *; *p* < 0.1 = ^(^*^)^.

The participants’ gaze behavior on the claims was first analyzed with descriptive methods. Hereafter, it was tested for differences in the frequencies of purchases of products labeled with a nutrition, health, or taste claim with non-parametric chi-square tests. To examine the relationships between the constructs depicted in the theoretical framework (Figure 2), structural equation modeling (SEM) was applied. As introduced in Table 2, several of these constructs were dichotomous. The software WarpPLS 6.0 (www.warppls.com) was used because it utilizes a partial least squares (PLS) regression procedure to model non-linearity among the constructs, irrespective of their measurement; metric, nominal, or even dichotomous [106,107,108]. PLS-SEM uses a variance-based algorithm (versus a covariance analysis algorithm) which maximizes the explained variance of the dependent constructs in the path model [109].

## 4. Results

### 4.1. Gaze Duration on Claims

Participants spent on average 0.95 s (SD = 0.76 s) looking at the taste claim, 1.16 s (SD = 0.91 s) looking at the nutrition claim, and 1.37 s (SD = 1.19 s) looking at the health claim. Paired sample *t*-tests revealed that the gaze durations were significantly different between the claim types. A possible explanation is that the tested claims were different in length, with the taste claim being the shortest and the health claim being the longest. Research on eye tracking has shown that consumers cognitively process information in that moment they are looking at it [70,71,75,76,110]; so the differences in gaze durations across the claim types might be attributed to different levels of complexity of information processing.

### 4.2. Purchase Decision

With chi-square tests, whether the share of purchases of orange juices with a specific claim type was significantly higher or lower than the so-called expectancy value was analyzed, which represents the assumption for a specific claim type not having an effect on the purchase decision. This value is 33.33% because the three claim types were equally present in each product set. Orange juices labeled with the nutrition claim (40.8%) were bought significantly more often (χ² (1) = 4.1407, *p* = 0.0419). However, the shares of purchases for orange juices labeled with the taste claim (30.6%) or the health claim (28.7%) were not significantly different from the expectancy value.

### 4.3. Structural Equation Model

The prerequisites for running the SEM analysis were met: Collinearity among the latent constructs was low and all estimated measurement errors were lower than their estimated corresponding composite weights. After the SEM was run, the obtained indices confirmed the overall good fit of the model with the data (Table 4). It is of special interest that the values for the average block variance inflation factor (AVIF) and average full collinearity variance inflation factor (AFVIF) were both far below 3.3, thus fulfilling the recommendation for models with many single-indicator variables [111]. To check the internal consistency of the variables, composite reliabilities were used, as they were deemed to be an appropriate approach for estimating the reliabilities in a PLS-based structure equation model [112,113,114]. As shown in Table 5 and Table 6, all variables had acceptable internal consistency. Furthermore, the square root of the average variance extracted (AVE) for each variable exceeded the correlations between one variable with the other variables, thus showing discriminant validity [115].

Regarding the purchase of products labeled with a nutrition claim, the analysis provided the following results. Nutrition knowledge and health motivation had significant positive effects on the gaze on the nutrition claim and explained 11.2% of its variance (Table 7), i.e., consumers with higher nutrition knowledge and higher health motivation looked at the nutrition claim longer. However, neither consumer characteristic showed an effect on the purchase decision. Neither the direct effect nor the total effect of nutrition knowledge and health motivation on purchase was significant (Table 8). Gaze, by contrast, had a significant positive effect on purchase, in that the longer a consumer gazed at the nutrition claim, the more likely the product with a nutrition claim was bought. Gaze accounted for 4.6% of the variance observed in the purchase decision for products with a nutrition claim. The model shows that gaze behavior is neither a mediator between nutrition knowledge and purchase decision, nor between health motivation and purchase decision. When it comes to the product attributes included in the model, perceived healthiness and tastiness both had a significant positive effect, whereas price had a significant negative effect on the purchase decision. Together, the product attributes explained 25.1% of the variance in the purchase of products with nutrition claims. Interestingly, brand had no effect on purchase. In total, consumer characteristics and product attributes were able to explain 30.5% of the variance in the variable ‘purchase of products with a nutrition claim’. The calculated model and its path coefficients are depicted in Figure 2.

Regarding the purchase of products labeled with a health claim, the model could only explain 8.1% of the variance. The same significant influencing factors were identified, with the exception that perceived healthiness had no significant influence on the purchase of products with a health claim (Table 8 and Table 9).

### 4.4. Additional Results

We carried out additional analyses to provide further insights into the results of the SEM. The finding that nutrition knowledge and health motivation had a positive influence on gaze duration on both types of claims led to the assumption that more knowledgeable and health-motivated consumers might seek more product information in general, and thus look longer at product packages. A correlation analysis with the variable ‘total gaze duration on all three packages’ confirmed this assumption for nutrition knowledge (*r* = 0.260, *p* < 0.01) and health motivation (*r* = 0.250, *p* < 0.01).

Since previous studies have suggested more knowledgeable and health-motivated consumers might be more skeptical about nutrition and health claims, we also tested this relationship. In the questionnaire, the participants were asked to rate the credibility of the given nutrition and health claims on a 7-point Likert scale. However, the correlation analysis found no significant relationships, neither with nutrition knowledge (nutrition claim: *r* = −0.096; health claim: *r* = 0.007; all *p*-values > 0.1), nor with health motivation (nutrition claim: *r* = −0.039; health claim: *r* = 0.136; all *p*-values > 0.1)

## 5. Discussion and Conclusions

The purpose of this study was to investigate the role of consumers’ nutrition knowledge and health motivation together with gaze behavior in purchase decisions for products with nutrition and health claims.

### 5.1. Influence of Consumer Characteristics

To measure the influence of nutrition knowledge and health motivation on gaze duration and purchase behavior, a structural equation model was used. Its theoretical framework was based on the elaboration likelihood model: Consumers with higher nutrition knowledge and higher health motivation will contemplate nutrition and health claims to a higher degree. With the use of eye tracking, this study was able to show that consumers with higher nutrition knowledge and higher health motivation looked at nutrition and health claims to a greater extent when making a purchase decision compared to other consumers.

Since there is a strong relationship between visual attention and elaboration [123], one can say that higher attention means a higher elaboration of certain information. Therefore, the results obtained by this study are in line with the Elaboration Likelihood Model, in that knowledge and motivation led to a different elaboration. However, the results of the present study do not support the suggestions of previous researchers that health motivation and nutrition knowledge might influence the choice of food products labeled with nutrition or health claims. Consumers with higher health motivation and higher nutrition knowledge were indeed more interested in the nutrition and health claims. Also, they looked longer at the packages in general. These consumers might have understood that all product alternatives—no matter the type of claim—offered just the same nutritional composition and health benefits. Previous researchers suggested that one explanation could be that these consumers were too skeptical about the nutrition and health claims [9,90,124]. However, in the present study, health motivation and nutrition knowledge did not correlate with the credibility of the claims, nor did they have an effect on the purchase of products labeled with claims. Perhaps higher motivation or knowledge does not always translate into a change in purchase behavior, let alone eating behavior, as reported in other research [85,125].

The structural equation model further showed that an increase in visual attention on the nutrition claim (health claim) led to an increase in the purchase likelihood of the product labeled with the nutrition claim (health claim). This is in line with previous eye tracking research that determined that consumers who look at a product package or at its elements longer will be more likely to choose this product [44,53,55,56]. The purchase decision for products with a nutrition claim (health claim) was explained to 4.6% (1.4%) by gaze on the respective claim.

### 5.2. Influence of Product Attributes

The present analysis showed that, besides gaze behavior, product attributes also influenced the purchase decision. In the nutrition claim model, price had the greatest effect on the purchase decision (15.5%), followed by perceived healthiness (6%) and perceived tastiness (3.6%). In the health claim model, price had the greatest effect on the purchase decision (3.7%), followed by perceived tastiness (3.2%). This result is consistent with previous research findings, suggesting that foods are chosen mainly based on price, taste, and healthiness [59,126,127,128,129,130]. Often enough, price is the most decisive aspect for food purchases, especially in Germany [131,132,133].

### 5.3. Implications

The findings of the study have several implications for policy makers and marketers alike. Consumers with high nutrition knowledge and health motivation looked at the nutrition and health claims longer but did not buy these products more often than less knowledgeable and motivated consumers did. For food companies, the present findings imply that, to target consumer groups with higher health motivation and nutrition knowledge, it is not enough to label products with nutrition and health claims. These consumers might critically evaluate such claims and rely on other product attributes when deciding which food product to choose. In addition, health claims were less preferred than nutrition claims in our study, so marketers planning to introduce a health claim are advised to pretest whether consumers would actually prefer a health claim or nutrition claim on that particular product.

The findings of the present study showed that nutrition claims have an effect on food choice. In the experiments, the participants could choose among three products with identical nutrition profiles, but the nutrition claim product was still clearly preferred. Even consumers with higher nutrition knowledge and health motivation preferred the nutrition claim product as much as less knowledgeable and motivated consumers did. Apparently, the nutrition information on the back of the package was not enough to make consumers realize the three products were identical. Policy makers should consider introducing mandatory standardized nutrition information on the package front. The format of the nutrition information should be as easy to understand as the nutrition claim tested in our study.

### 5.4. Mixed Methods Approach

The originality of this study lies in the mixed methods approach. The two consumer characteristics ‘nutrition knowledge’ and ‘health motivation’ were measured with a questionnaire, the purchase decision in a close-to-realistic purchase simulation, while eye tracking glasses recorded the gaze behavior. The nutrition and health claims were unobtrusively incorporated on real food packages which were placed on a shopping shelf. The claims were not forcefully exposed to the participants, unlike this widespread practice in previous studies within this research area [25,134]. The combination of eye tracking and questionnaire data led to a better understanding of the influence of consumer characteristics on the gaze and purchase behavior for products with nutrition and health claims than did the use of only one method on its own [52].

## 6. Limitations and Future Research

Unknown food brands were used in the purchase simulations to avoid consumers purchasing their favorite brands or relying on previous experiences, since grocery shopping is usually a low-involvement situation [53]. However, this limits the generalizability of the results to shopping decisions without well-known brands. In an experiment with familiar brands and packaging designs, the consumers might have looked differently at the products and the claims. In future research, the study design could be expanded to include familiar brands in the testing of nutrition and health claims.

The nutrition and health claims for orange juice tested in the present study referred to vitamin C which German consumers are very familiar with [135]. Vitamin C is a natural component of orange juice. Previous studies showed that familiarity with the product and the ingredient mentioned in the claim, as well as a natural fit of the ingredient–product combination, has a positive influence on the effect of nutrition and health claims on preferences and purchases [136,137,138,139]. Nutrition and health claims about vitamins and their reference to the benefits for the immune system are among the most preferred [32] and widely used claims in the European Union [4,8,26]. Therefore, it needs to be recognized that the results of the present study are limited in its generalizability to products and ingredients consumers are very familiar with. Future research could test different products or a combination of novel products and claims.

The explanatory power of the study would have been higher if more different product categories had been included. However, the preparation of the data collected with a head-mounted eye tracking system is very labor-intensive. Previous researchers have pointed to this issue as the main determinant for the limited sample size of studies with a head-mounted eye tracking system [50,97]. In the future, once computer software will carry out the nowadays labor-intensive stage of data preparation, it will be possible to conduct larger experiments with more products.

Lastly, previous research has suggested that the effect of nutrition and health claims on preferences and purchases cannot easily be transferred between different countries [11,21,137]. Future studies could test the robustness of the results obtained in this study in different countries.

## Figures and Tables

**Table 1:** Nutrition and health claims used in the study.

	Orange Juice
Nutrition claim	Rich in vitamin C
Health claim	Vitamin C contributes to the normal function of the immune system

**Table 2:** Overview of the constructs and their indicators in the model.

Construct	Indicator
Nutrition knowledge	Indicator 1: Knowledge about the calorie content of various foods. Measured with three questions, resulting in a metric indicator ranging from 1 to 3. Indicator 2: Knowledge about the nutrient composition of various foods. Measured with five questions, resulting in a metric indicator ranging from 1 to 5. Indicator 3: Knowledge about the relationship between food intake and disease. Measured with two questions, resulting in a metric indicator ranging from 1 to 2.
Health motivation	Each of the five indicators were measured on a 7-point Likert scale with 1 = strongly disagree, 7 = strongly agree. Indicator 1: I pay a lot of attention to healthy foods. Indicator 2: A healthy diet is very important to me. Indicator 3: I pay close attention to the health benefits of food. Indicator 4: I always eat what I want without worrying about the health of my diet. Indicator 5: I inform myself very often about nutrition.
Gaze on claim	Indicator 1: ‘Dwell time’ on specific claim, measured in seconds. Indicator 2: ‘Net dwell time’ on specific claim, measured in seconds. Indicator 3: ‘Visual intake time’ on specific claim, measured in seconds. Indicator 4: ‘Visual intake count’ on specific claim, measured in counts.
Perceived healthiness of product	The two indicators were measured separately for each of the three products tested in the purchase simulation Indicator 1: How healthy are the orange juices you just looked at? 7-point Likert scale with 1 = very unhealthy to 7 = very healthy Indicator 2: How healthy are the offered orange juices compared to the orange juices you are familiar with? 7-point Likert scale with 1 = much unhealthier to 7 = much healthier
Perceived tastiness of product	The indicator was measured separately for each of the three products tested in the purchase simulation Indicator: How do you rate the taste of the offered orange juices? 7-point Likert scale with 1 = very bad taste to 7 = very good taste.
Price for product	The indicator is a metric variable ranging from €1.09 to €.1.49
Brand 1 for product	The indicator is a dichotomous variable, representing the purchase of brand 1 vs. the two other brands.
Brand 2 for product	The indicator is a dichotomous variable, representing the purchase of brand 2 vs. the two other brands.
Purchase product	The indicator is a dichotomous variable, representing the purchase of a product with the specific claim vs. the purchase of a product with the two other respective claims.

**Table 3:** Socio-demographic characteristics of the sample.

Characteristic	Description	Sample	Population City *
Age (*n* = 152)	Average	41.2	42.6
	18–44	53.9%	48.8%
	45–64	34.2%	33.8%
	>65	11.9%	17.4%
Sex (*n* = 156)	Female	49.4%	51.0%
	Male	50.6%	49.0%
Households (*n* = 156)	Average number of household members	1.9	1.9
	One-person households	48.7%	51.9%
	Households with children	25.6%	17.2%
	Households with three or more children	3.2%	12.5%
Household income (*n* = 156)	Average monthly disposable household income	1796.8 €	1821.5 €

* Source: Kassel—Department of Statistics [105].

**Table 4:** Model fit and quality indices.

Index	Value	Criteria
Average path coefficient (APC)	0.137 (*p* = 0.020)	*p*-values lower than 0.05 are recommended [116]
Average R-squared (ARS)	0.142 (*p* = 0.017)	
Average adjusted R-squared (AARS)	0.114 (*p* = 0.037)	
Average block variance inflation factor (AVIF)	1.303	Values lower than 3.3 are recommended [117]
Average full collinearity variance inflation factor (AFVIF)	2.281	
Tenenhaus GoF (GoF) – measure of the model’s explanatory power	0.359	small ≥0.1, medium ≥0.25, large ≥0.36 [114,118]
Sympson’s paradox ratio (SPR)	0.850	Values higher than 0.7 are recommended [119,120,121]
R-squared contribution ratio (RSCR)	0.988	Values higher than 0.9 are recommended [111]
Statistical suppression ratio (SSR)	0.750	Values higher than 0.7 are recommended [122]
Nonlinear bivariate causality direction ratio (NLBCDR)	0.975	Values higher than 0.7 are recommended [111]

**Table 5:** Correlations, composite reliabilities, Cronbach α, and average variances extracted—nutrition claim.

Variable	CR	Cr α	1	2	3a	4a	5a	6a	7a	8a	9a
1. Nutrition knowledge	0.729	0.712	(0.691)								
2. Health motivation	0.902	0.860	0.058	(0.808)							
3a. Gaze on claim—NC	0.995	0.993	0.145 ^(^*^)^	0.236 **	(0.99)						
4a. Healthiness—NC	0.724	0.619	−0.182 *	−0.212 **	−0.243 **	(0.851)					
5a. Tastiness—NC	1	1	−0.052	−0.126	−0.243 **	0.64	(1)				
6a. Price—NC	1	1	0.114	−0.124	−0.083	0.087	0.035	(1)			
7a. Brand 1—NC	1	1	0.043	−0.01	−0.199 *	0.109	0.152 ^(^*^)^	0.052	(1)		
8a. Brand 2—NC	1	1	−0.061	−0.064	0.069	−0.024	−0.024	0.003	−0.522	(1)	
9a. Purchase—NC	1	1	−0.042	0.061	0.188 *	0.244 **	0.238 **	−0.39	−0.02	0.078	(1)

Significance *p* < 0.01 = **; *p* < 0.05 = *; *p* < 0.1= ^(^*^)^; Square roots of average variances extracted (AVE) are shown on diagonal. NC = Nutrition claim.

**Table 6:** Correlations, composite reliabilities, Cronbach α, and average variances extracted—health claim.

Variable	CR	Cr α	1	2	3b	4b	5b	6b	7b	8b	9b
1. Nutrition knowledge	0.729	0.712	(0.691)								
2. Health motivation	0.902	0.860	0.058	(0.808)							
3b. Gaze on claim—HC	0.993	0.990	0.163 *	0.146 ^(^*^)^	(0.986)						
4b. Healthiness—HC	0.754	0.675	−0.101	−0.205 *	−0.116	(0.869)					
5b. Tastiness—HC	1	1	−0.035	−0.114	−0.054	0.658	(1)				
6b. Price—HC	1	1	−0.042	0.087	−0.048	−0.059	0.034	(1)			
7b. Brand 1—HC	1	1	−0.031	0.012	−0.065	0.158 *	0.193 *	0.017	(1)		
8b. Brand 2—HC	1	1	0.146 ^(^*^)^	0.02	0.235 **	0.083	0.042	−0.001	−0.529	(1)	
9b. Purchase—HC	1	1	0.065	−0.035	0.124	0.052	0.147 ^(^*^)^	−0.191 *	0.021	0.034	(1)

Significance *p* < 0.01 = **; *p* < 0.05 = *; *p* < 0.1= ^(^*^)^; Square roots of average variances extracted (AVE) are shown on diagonal. HC = Health claim.

**Table 7:** Path coefficients and their effect sizes—nutrition claim.

	Gaze on Nutrition Claim	Purchase Decision for Product with Nutrition Claim
1. Nutrition knowledge	0.240 (0.063) ***	0.023 (0.001)
2. Health motivation	0.209 (0.049) **	0.031 (0.002)
3a. Gaze on claim—NC		0.245 (0.046) ***
4a. Healthiness—NC		0.248 (0.060) ***
5a. Tastiness—NC		0.152 (0.036) *
6a. Price—NC		–0.398 (0.155) ***
7a. Brand 1—NC		0.051 (0.001)
8a. Brand 2—NC		0.102 (0.008)

Significance *p* < 0.001 = ***; *p* < 0.01 = **; *p* < 0.05 = *; Effect sizes are shown in brackets.

**Table 8:** Total effects and their effect sizes—nutrition and health claim.

	Purchase Decision for Product with Nutrition Claim	Purchase Decision for Product with Health Claim
1. Nutrition knowledge	0.082 (0.003) n.s.	0.058 (0.004) n.s.
2. Health motivation	0.082 (0.005) n.s.	−0.012 (0.001) n.s.

n.s. = not significant; Effect sizes are shown in brackets.

**Table 9:** Path coefficients and their effect sizes—health claim.

	Gaze on Health Claim	Purchase Decision for Product with Health Claim
1. Nutrition knowledge	0.174 (0.034) *	0.038 (0.002)
2. Health motivation	0.172 (0.033) *	−0.032 (0.001)
3b. Gaze on claim—HC		0.114 (0.014) ^(^*^)^
4b. Healthiness—HC		−0.094 (0.005)
5b. Tastiness—HC		0.218 (0.032) **
6b. Price—HC		−0.194 (0.037) **
7b. Brand 1—HC		0.009 (0.001)
8b. Brand 2—HC		0.005 (0.001)

Significance *p* < 0.01 = **; *p* < 0.05 = *; *p* < 0.1= ^(^*^)^; Effect sizes are shown in brackets.
